# Supplementary material for: Learning curve and surgical outcome of robotic assisted colorectal surgery with ERAS program
Source: Sci Rep. 2022 Nov 29;12:20566. doi: 10.1038/s41598-022-24665-w (PMC9709162; doi:10.1038/s41598-022-24665-w)
Supplement: Supplementary file 1 — Supplementary Information 1. [file 41598_2022_24665_MOESM1_ESM.zip › LSC Raw data-Ñ[▒K/141/O_ñw╝╨░O▒Kñσ.pdf]

正式摘要(電子簽章：000000227498332)

請選擇...reporter

臺中榮民總醫院

Operation Note

[redacted]

- 手術日期：2021/10/13
- 手術醫師：VS 林俊余，R 林志安，R 林順鴻
- Anesthesia：GE

Diagnosis

- Pre-operative diagnosis : rectum ca
- Post-operative diagnosis : rectum adenocarcinoma with peranal excision s/p laparoscopic LAR on 2021/10/13

Surgical Indication

- To remove primary tumor and for tumor staging

Operation

- 1. Laparoscopic low anterior resection
  2. Intraoperative colonoscopy : (-)
  3. High ligation (+) : At IMA root. IMV and left colic artery were also ligated.
  4. EndoGIA (60-4.1 ; Green) X4 + EEA (29 mm) were used
  5. Proximal cut end: >10cm, distal cut end: 0.5cm
  6. One 15 Fr. Hemovac drainge tube was placed from LLQ of abdomen and inserted into the abdominal cavity
  7. The colon was not taken down from the splenic flexure site
  7. both circular donut intack, air leak test negative
  8. loop ileostomy was done over RLQ

Finding

- 1. A 0.5*0.5cm scaring tissue noted over distal end of the rectum, margin not involved grossly
  2. Palpable LN over the mesorectum
  3. There was no obvious visible liver metastasis, no visible peritoneal seeding.
  4. Bilateral doughnuts : intact ; Air leak test: Done and no air leak was found
  5. During end to end anastmosis, suspected serosa tear of the distal end stump of rectum was noted.
  6. Hematuria noted during operation, left ureter checked intact
  7. margin: distal 0.5cm

Procedure

- 1. General endotracheal anesthesia was induced and then the patient was placed in modified Trendelenburg lithotomy position. Preoperative antibiotics were given. A Foley catheter and a nasogastric tube were placed. The abdomen was prepared and draped in standard sterile fashion.
  2. A small vertical incision was made right next to the umbilicus and a 12 mm trocar was inserted into the peritoneal cavity by open method. Peumoperitoneum was established with CO2 inflation. Laparoscopy was inserted and then another 12 mm trocar was inserted over RLQ. Extra three 5 mm trochars were inserted over RUQ, LUQ and LLQ of abdomen. The operative findings were mentioned as above.
  3. The small bowel was inspected and retracted to the right by grasper. The sigmoid colon was then held by grasper and the peritoneum overlying the mesentery was then scored with electrocautery. The inferior mesentery artery was identified, ligated below the left colic artery with endoclips, and transected, so was inferior mesentery vein. Dissection was done above the Gerota's fascia and toward the peritoneal attachments.

- 4. The arcade vessels to the sigmoid colon were also divided. Distal descending colon and upper rectum were mobilized. Totally Four linear staplers (EndoGIA 60-4.1) were used to divide the rectum.
  5. Incision was further extended from the RLQ 5 mm trochar wound. The proximal colon was then taken out for carefully inspection, and to make sure it has an excellent blood supply. The mesenteric fat was gently cleared from the terminal 2-3 mm of the bowel ends.
  6. The field was isolated with moist pad and the bowel opened and sized. It was determined that a 29 mm circular stapler could be accommodated. A pursestring suture of 2-0 prolene was placed, the anvil was inserted, and then the pursestring suture was tied.
  7. The anal canal was irrigated with distil water and then the circular stapler was introduced through the anus and pierced through the mid-portion of the stapler line.
  8. The proximal bowel with anvil in place was engaged with the circular stapler and the stapler was then fired. Two complete donuts were obtained and the distal one was submitted to pathology for margin confirmation.
  9. The anastomosis was tested for leaks with irrigation solution and air introduced through the rectum and was found to be patent and intact.
  10. The possible bleeder was then checked thoroughly.
  11. 15Fr hemovac drain was placed over LLQ and place over the pelvis.
  12. Terminal ileum was identified and was then dragged out from the RLQ carefully and a plastic bridge (Rod) was placed via the window.
  13. The fascia of midline wound was closed with interrupted sutures of 1-O Vicryl. The fascia of the 12 mm trochar was also closed with 1-0 Vicryl. The LLQ extended wound was further closed with interrupted method via Num. 1 Vicryl.
  14. Skin were closed with interrupted 3-0 nylon.
  15. Loop ileostomy was matured with 4-0 monocryl
  15. The patient tolerated the procedure well and was brought to the POR in stable condition.
- Estimated blood loss: minimum
- 器械數量: 正確
- 紗布數量: 正確

Image

- PAINT
  - abdomen

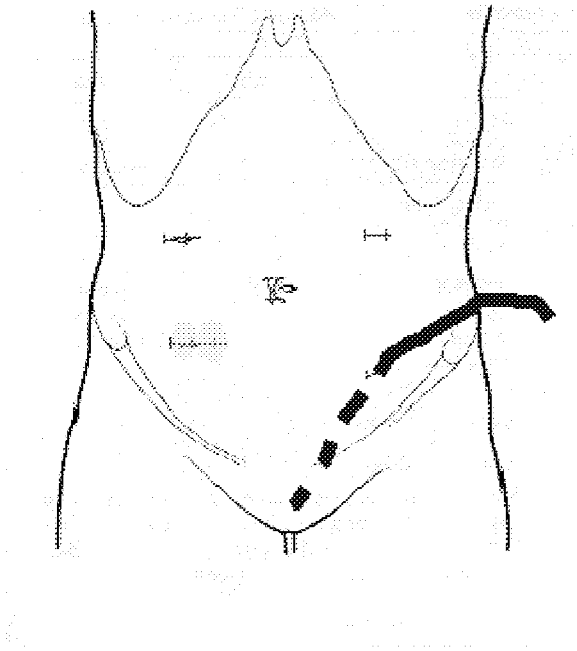

  - colon & rectum

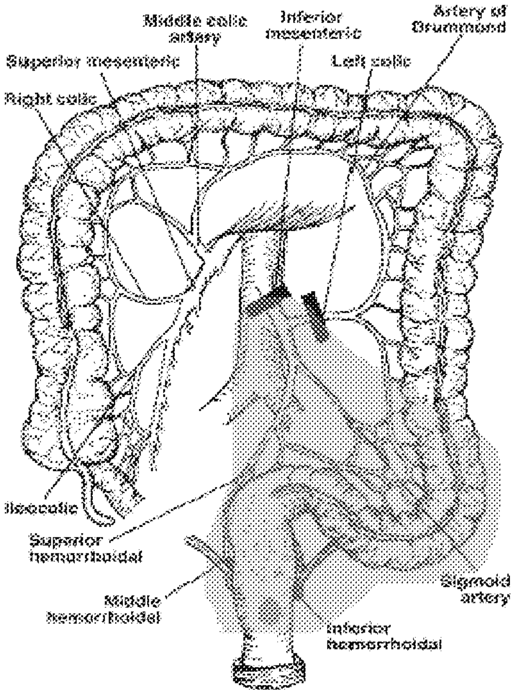

主治 2222H林俊余 / 醫師 2332G林志安

正式摘要(電子簽章：000000227498332)

請選擇...reporter

doctorRank:B
